# Urinary hepcidin is potentially a marker of systemic infection rather than inflammation, in the setting of preserved renal function

**DOI:** 10.1186/cc10377

**Published:** 2011-10-27

**Authors:** NJ Glassford, AG Schneider, G Eastwood, L Peck, H Young, M Westerman, V Ostland, R Bellomo

**Affiliations:** 1 Department of Intensive Care, Austin Hospital, VIC, Australia; 2 Intrinsic LifeSciences LLC, La Jolla, CA, USA

## Introduction

Urinary proteomics have recently identified hepcidin, a key regulator of iron homeostasis, as a potential marker of tubular stress [1]. It appears to be released in response to situations that predispose to acute kidney injury (AKI), and greater concentrations of hepcidin in the blood and in the urine have been associated with reduced risk of AKI [2]. Catalytic iron is a biologically plausible mechanism for the development of AKI as a consequence of tubular oxidative stress [3]. The relationship between serum creatinine, urinary hepcidin and CRP may help define whether urinary hepcidin is more likely to reflect systemic inflammation or renal events. The relationship in septic patients has not yet been described. Patients with SIRS, oliguria and a 25 μmol/l increase from baseline creatinine are known to be at an increased risk of AKI [4]. We sought to determine if hepcidin correlated more strongly with CRP or creatinine in these patients with a diagnosis of sepsis and those without.

## Methods

Patients meeting the inclusion criteria within 48 hours of admission had their CRP, urinary hepcidin, and serum and urinary creatinine measured. The strength of the relationship between serum creatinine or CRP and urinary hepcidin corrected for urinary creatinine was determined using Spearman's rank correlation coefficient.

## Results

We enrolled 103 patients between 31 August 2010 and 17 November 2010; 22 of whom had an APACHE III diagnosis of sepsis. Serum creatinine only correlated weakly with direct and inverse urinary hepcidin measurements in septic and nonseptic patients alike. However, there was a moderately strong correlation between CRP and urinary hepcidin in septic patients, a relationship not demonstrated in the nonseptic group (Table 1).

**Table 1:** Relationships between hepcidin, creatinine and CRP

	Correlation			
	
	Nonseptic (*n *= 81)		Septic (*n *= 22)	
	
Variable	Serum Cr	CRP	Serum Cr	CRP
Urinary hepcidin	-0.272	0.204	-0.225	0.506
	(*P *= 0.013)	(*P *= 0.064)	(*P *= 0.314)	(*P *= 0.016)
1/urinary hepcidin	0.287	-0.19	0.225	-0.506
	(*P *= 0.009)	(*P *= 0.087)	(*P *= 0.314)	(*P *= 0.016)
Urinary hepcidin corrected for urinary creatinine	-0.146	0.241	-0.276	0.418
	(*P *= 0.191)	(*P *= 0.029)	(*P *= 0.227)	(*P *= 0.06)
1/urinary hepcidin corrected for urinary creatinine	0.158	-0.228	0.276	-0.418
	(*P *= 0.159)	(*P *= 0.041)	(*P *= 0.227)	(*P *= 0.06)

## Conclusion

Hepcidin is only weakly inversely correlated with serum creatinine. A stronger relationship exists between hepcidin and CRP in septic patients, suggesting that hepcidin may primarily be a marker of infection that is filtered in the urine when the glomerular filtration rate (GFR) is preserved and filtered in lower amounts when the GFR is lost. That this relationship is not replicated in nonseptic patients with clinical evidence of SIRS suggests that the underlying pathophysiological processes are different. Further investigation of the natural history of AKI and biomarker release is warranted.
